# Intravitreal vs. subtenon triamcinolone acetonide for the treatment of diabetic cystoid macular edema

**DOI:** 10.1186/1471-2415-8-5

**Published:** 2008-03-17

**Authors:** Mauro Cellini, Alberto Pazzaglia, Eugenio Zamparini, Pietro Leonetti, Emilio C Campos

**Affiliations:** 1 Department of Surgery and Transplant "A. Valsalva", Ophthalmology Service (Chief: Prof. E.C. Campos), University of Bologna, Italy

## Abstract

**Background:**

To assess the efficacy of the intravitreal (IVT) injection of Triamcinolone Acetonide (TA) as compared to posterior subtenon (SBT) capsule injection for the treatment of cystoid diabetic macular edema.

**Methods:**

Fourteen patients with type II diabetes mellitus and on insulin treatment, presenting diffuse cystoid macular edema were recruited. Before TA injection all focal lakes were treated by laser photocoagulation. In the same patients one eye was assigned to 4 mg IVT injection of TA and the fellow eye was then treated with 40 mg SBT injection of TA. Before and one, three and six months after treatment we measured visual acuity with ETDRS chart as well as thickness of the macula with optical coherence tomography (OCT) and intraocular pressure (IOP).

**Results:**

The eyes treated with an IVT injection displayed significant improvement in visual acuity, both after one (0.491 ± 0.070; p < 0.001) and three months (0.500 ± 0.089; p < 0.001) of treatment. Significant improvement was displayed also in eyes treated with an SBT injection, again after one (0.455 ± 0.069; p < 0.001) and three months (0.427 ± 0.065; p < 0.001). The difference between an IVT injection (0.809 ± 0.083) and SBT injection (0.460 ± 0.072) becomes significant six months after the treatment (p < 0.001).

Macular thickness of the eyes treated with IVT injection was significantly reduced both after one (222.7 ± 13.4 μm; p < 0.001) and after three months (228.1 ± 10.6 μm; p < 0.001) of treatment. The eyes treated with SBT injection displayed significant improvement after one (220.1 ± 15.1 μm; p < 0.001) and after three months (231.3 ± 10.9 μm; p < 0.001). The difference between the eyes treated with IVT injection (385.2 ± 11.3 μm) and those treated with SBT injection (235.4 ± 8.7 μm) becomes significant six months after the treatment (p < 0.001).

Intraocular pressure of the eyes treated with IVT injection significantly increased after one month (17.7 ± 1.1 mm/Hg; p < 0.020), three (18.2 ± 1.2 mm/Hg; p < 0.003) and six month (18.1 ± 1.3 mm/Hg; p < 0.007) when compared to baseline value (16.1 ± 1.402 mm/Hg). In the SBT injection eyes we didn't display a significant increase of intraocular pressure after one (16.4 ± 1.2 mm/Hg; p < 0.450), three (16.3 ± 1.1 mm/Hg; p < 0.630) and six months (16.2 ± 1.1 mm/Hg; p < 0.720) when compared to baseline value (16.2 ± 1.3 mm/Hg).

**Conclusion:**

The parabulbar subtenon approach can be considered a valid alternative to the intravitreal injection.

**Trial registration:**

Current Controlled Trials **ISRCTN67086909**

## Background

Diabetic macular edema is one of the leading causes of diabetes induced visual impairment and affects one third of diabetic patients with disease duration of twenty years or more [1]. Many studies, including the Early Treatment Diabetic Retinopathy Study (ETDRS) have demonstrated that macular photocoagulation is effective for the treatment of macular edema [2,3] but does not usually restore vision loss occurring before treatment [2,4]. Laser photocoagulation, however, only has a moderate effect in preventing further visual loss in about 50% of patients [2,4].

Recently, there have been many reports of the effectiveness of intravitreal triamcinolone acetonide (TA) for the treatment of diffuse macular edema, refractory to laser treatment [5,6].

Intravitreal triamcinolone injections are however associated with many ocular complications (i.e. ocular hypertone, endophthalmitis, intraocular hemorrhages, detachment of the retina) [5,7,6,9]. Parabulbar subtenon injection of steroids appears to offer a good alternative for the treatment of diabetic macular edema and intermediate uveitis [10,11]. This approach is less invasive than intravitreal injection and may deliver equivalent therapeutic quantities of TA to the retina [12].

The purpose of this study was to assess the efficacy of the intravitreal (IVT) injection of TA as compared to posterior subtenon (SBT) capsule injection for the treatment of cystoid diabetic macular edema.

## Methods

A total of 14 patients (28 eyes) were treated, 10 males and 4 females, aged between 61 and 74 years (mean 68.3), with type II diabetes mellitus and on insulin treatment.

All patients were phakic and showed a diffuse macular edema without retinal-vitreous traction. The patients were recruited among patients treated by the Ophthalmology Service of the S. Orsola-Malpighi Hospital, Bologna. Before enrolment, patients were informed of the procedures and the aim of the study and they signed a written consent form. The institutional ethics committee of the S. Orsola-Malpighi Hospital also approved the study. In all the patients the best corrected logarithm of the minimum angle of resolution (logMAR) visual acuity was assessed using the Early Treatment Diabetic Retinopathy Study (ETDRS) chart, as well as intraocular pressure (IOP) applanation tonometry and anterior and posterior segment biomicroscopy. Macular edema was defined by central thickening revealed with biomicroscopy using a 78-diopter non-contact lens and by diffuse fluorescein leakage on fluorescein angiography (FA). Macular thickness was measured by optical coherence tomography (OCT). All focal lakes previously were treated by laser photocoagulation.

Exclusion criteria included history of uveitis episodes, previous ocular surgery, glaucoma and ocular hypertension.

In the same patients one eye was assigned with a, random method generated by a computer to intravitreal (IVT) injection of TA. One week after the IVT treatment of the first eye, and after excluding the appearance of complications (i.e. hypertone, vitreous hemorrhage, endophthalmitis), the fellow eye was then treated with subtenon (SBT) injection of TA. Two days before the IVT injection of TA, to avoid post-operative hypertone, the patients were prescribed a systemic treatment with acetazolamide, 250 mg two times daily.

For the IVT injection, the patient was placed supine and we performed a surface anesthesia with topical 4% carbocaine followed by a preparation with 5% povidone iodine. A volume of 0.1 ml containing 4 mg preservative-free TA (Kenacort, Bristol-Myers Squibb, Sermoneta, Italy) was injected through the inferotemporal pars-plana (4.0 mm posterior to the limbus) using a 30-gauge needle. Indirect ophthalmoscopy was used to confirm correct intravitreal localization of the suspension. After the injection topical 0.3% Netilmicin ointment was prescribed.

For the posterior SBT injection, the patient was placed supine and after topical 0.4% oxybuprocaine surface anesthesia a 1 ml of a 40 mg/ml of triamcinolone acetonide (Kenacort, Bristol-Myers Squibb, Sermoneta, Italy) was given in the inferotemporal quadrant using a 27-gauge needle on 2.5-ml syringe. The patients were direct to look in the extreme superonasal field of gaze. The conjunctiva and the Tenon's capsule were penetrated with the bevel of the needle toward the globe. The needle was advanced toward the macular area, taking care to remain in contact with the globe until the hub was firmly pressed against the conjunctival fornix and then the corticosteroid was slowly injected. After injection topical 0.3% Netilmicin ointment was prescribed.

**Figure 1:**
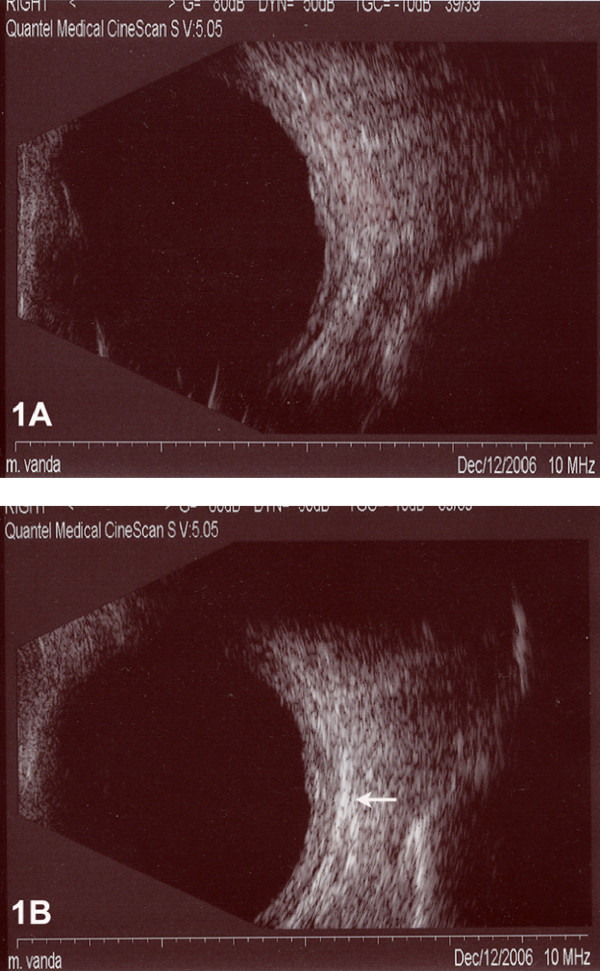
**Echographic images before and after subtenon triamcinolone injection**. Echographic B-scan image before (A) and after (B) posterior subtenon triamcinolone acetonide injection where the lucency (white arrow) representing repository corticosteroid.

For each patient in the group of SBT a B-scan examination was performed before and immediately after the injection to show the lucency in subtenon space representing the repository triamcinolone acetonide just in the macular area (Fig 1).

Subsequently, one, three and six months after treatment visual acuity and IOP were measured in the patients as well as thickness of the retinal macula with OCT.

The data were statistically evaluated using the Wilcoxon signed rank test and a p < 0.05 was considered significant. Data are presented as means ± standard deviation (SD). The analysis was performed using SSI (version 11, Systat Software Inc., San Jose, California, USA) for Macintosh.

## Results

The mean visual acuity before triamcinolone acetonide injection and after one, three and six months after are showed in Table 1. The eyes treated with an IVT injection displayed significant improvement in visual acuity, both after one month (0.491 ± 0.070; p < 0.001) and after three months (0.500 ± 0.089; p < 0.001) of treatment when compared to the baseline values (0.836 ± 0.112). Significant improvement was displayed also in eyes treated with an SBT injection, again after one month (0.455 ± 0.069; p < 0.001) and after three months (0.427 ± 0.065; p < 0.001) when compared to the baseline values (0.864 ± 0.103). The difference of visual acuity between eyes treated with an IVT injection and those treated with an SBT injection becomes significant six months after the treatment (p < 0.001). The eyes treated with IVT in fact display significant worsening of visual acuity (0.809 ± 0.083) whereas eyes treated with SBT maintain visual acuity improvement (0.460 ± 0.072).

**Table 1:** Visual acuity before and after IVT and SBT triamcinolone injection. Visual acuity with log/MAR resolution in the intravitreal and posterior subtenon injected eyes at baseline and at 1, 3 and 6 months after triamcinolone acetonide injection.

	IVT Visual Acuity	SBT Visual Acuity	p < 0.05
Baseline	0.836 ± 0.112	0.864 ± 0.103	0.625
1 month	0.491 ± 0.070	0.455 ± 0.069	0.161
3 months	0.500 ± 0.089	0.427 ± 0.065	0.070
6 months	0.809 ± 0.083	0.460 ± 0.072	0.001

**Figure 2:**
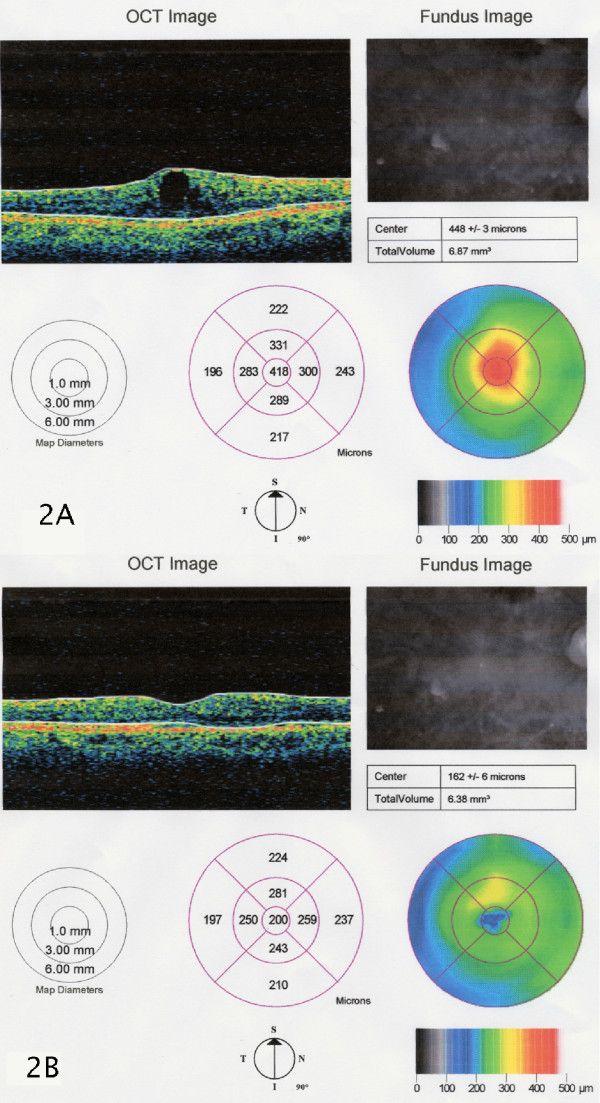
**OCT images before and after subtenon triamcinolone injection**. Optical coherence tomography map of diabetic cystoid macular edema before (A) and six months after (B) posterior subtenon triamcinolone acetonide injection.

The macular thickness before triamcinolone acetonide injection and after one, three and six months are showed in Table 2. The macular thickening of the eyes treated with IVT injection is concerned, thickness was significantly reduced both after one month (222.7 ± 13.4 μm; p < 0.001) and after three months (228.1 ± 10.6 μm; p < 0.01) when compared to the baseline values 386.3 ± 12.4 μm. The eyes treated with SBT injections displayed significant improvement after one month (220.1 ± 15.1 μm; p < 0.001) and after three months (231.3 ± 10.9 μm; p < 0.001) of treatment when compared to the baseline values of 384.1 ± 18.9 μm. Here too the difference in retinal macular thickness of the eyes treated with IVT (385.2 ± 11.3 μm) and those treated with SBT (235.4 ± 8.7 μm) becomes significant six months after the treatment (p < 0.001). Fig 2 illustrates the changes in the OCT images of a representative patient in the SBT injection group.

**Table 2:** Macular thickness before and after IVT and SBT triamcinolone injection. Central macular thickness (μm) in the intravitreal and posterior subtenon injected eyes at baseline and at 1, 3 and 6 months after triamcinolone acetonide injection.

	IVT Macular Thickness	SBT Macular Thickness	p < 0.05
Baseline	386.3 ± 12.4	384.1 ± 18.9	0.721
1 month	222.7 ± 13.4	220.1 ± 15.1	0.625
3 months	228.1 ± 10.6	231.3 ± 10.9	0.580
6 months	385.2 ± 11.3	235.4 ± 8.7	0.001

The mean intraocular pressure before triamcinolone acetonide injection and after one, three and six months are showed in Table 3. The IOP of the eyes treated with IVT injection was significantly increased after one month (17.7 ± 1.1 mm/Hg; p < 0.020), three months (18.2 ± 1.2 mm/Hg; p < 0.003) and six months (18.1 ± 1.320 mm/Hg; p < 0.007) when compared to baseline value (16.1 ± 1.4 mm/Hg) but none glaucoma medication was needed to control the IOP. The eyes treated with SBT injection displayed not significant increase of the IOP not only after one month (16.4 ± 1.2 mm/Hg; p < 0.450) but also after three (16.3 ± 1.1 mm/Hg; p < 0.630) and six months (16.2 ± 1.1 mm/Hg; p < 0.720) when compared to baseline value (16.2 ± 1.3 mm/Hg). The difference of IOP between eyes treated with an IVT injection and those treated with an SBT injection becomes significant at three (p < 0.026) and six (p < 0.030) months.

**Table 3:** Intraocular pressure before and after IVT and SBT triamcinolone injection. Intraocular pressure (mm/Hg) in the intravitreal posterior subtenon and injected eyes at baseline and at 1, 3 and 6 months after triamcinolone acetonide injection.

	IVT-IOP	SBT-IOP	p < 0.05
Baseline	16.1 ± 1.4	16.2 ± 1.3	0.140
1 month	17.7 ± 1.1	16.4 ± 1.2	0.062
3 months	18.2 ± 1.2	16.3 ± 1.1	0.026
6 months	18.1 ± 1.3	16.2 ± 1.1	0.030

## Discussion

This study demonstrates that three months after the intravitreal injection of TA and the subtenon injection of TA there is a statistically significant improvement in visual acuity and an equally significant reduction in retinal thickness. Six months after IVT the patients presented a recurrence of macular edema with loss of visual acuity whereas six months after SBT injection retinal thickness and visual acuity remained stable. After one, three and six months we observed a statistically significant rise of the IOP in the eyes treated with IVT injection whereas in the SBT injection group, no statistically significant variations of the IOP were found. None of patients developed cataract or needed anti-glaucoma drugs during the follow-up.

Macular edema is the main cause of loss of visual acuity in diabetic patients [13,14]. It may occur at any stage of the retinal disorder and is the most common cause of sight reductions in these subjects.

In the edema, the hemato-retinal barrier is damaged by an alteration in the tight junction between the retinal capillary endothelial cells and the pigmented epithelial cells with the consequent leakage of water and electrolytes in the retinal tissue [3,15-17].

As has been seen in numerous studies, including the Early Treatment Diabetic Retinopathy Study (ETDRS), macular photocoagulative treatment is effective in the treatment of clinically important macular edema [2,18,3].

Thus laser photocoagulation for macular edema, although successful in blocking further visual loss in 50% of patients, is unable to restore visual loss occurring prior to treatment [2,4]. Moreover, laser photocoagulation is not very effective in eyes with diffuse macular edema [19,20].

The extent of the restoration of the hemato-retinal barrier functioning following laser treatment is debated as many studies indicate an increase in the edema following laser photocoagulation [20-22] probably as a result of the release of proinflammatory molecules. Indeed, the initial clinical pattern of diabetic retinopathy, with vasodilatation, increased blood flow, tissue edema and an increase in the vascular permeability presents the characteristics of chronic inflammation. This hypothesis is supported by recent studies, which have highlighted the appearance of leukostasis in diabetes [23] with adhesion of activated molecules to the endothelium [24], increased production of prostacyclin [25], vascular endothelial growth factor (VEGF) and macrophagic cellular component [26].

Further support for the thesis of inflammation as one of the causes of onset of diabetic retinopathy is provided by experimental studies in animals which demonstrate how hyperglycemias not only causes an increase in the production of cycloxygenase-2 (COX2), through the activation of protein-kinase (PKC) [27,28], but also in prostaglandin synthetase (PGIS) [29] a specific enzyme in the synthesis of prostaglandin PGI2 [30]. Furthermore, recent studies have confirmed the important role of COX2 and the prostanoids in the onset of renal damage in patients with impaired glycemic control.

The fall in the prostacyclin levels only occurs in the advanced stage of diabetic microangiopathy. This is confirmed not only by the reduction in the blood PGI2 levels but also by the reduction in the PGE values observed at a vitreous level during proliferative diabetic retinopathy [31].

All these experimental and clinical data confirm the involvement of pro-inflammatory molecules that also cause a subclinical increase in the aqueous humor cells in the early stages of diabetic retinopathy [32].

Recent studies have shown that intravitreal injections of TA have a positive effect on those forms of diabetic macular edema that are refractory to retinal laser treatment [5,7,6]. The use of corticosteroids for the treatment of retinal edema is linked to their capacity to inhibit the initial arachidonic acid cascade, to determine a down-regulation of the cytokines and to attenuate the tearing of the hemato-retinal barrier [7,15,33].

The use of intravitreal TA is not however without risks [6,12]. The main complications are endophthalmitis, intraocular hemorrhages, detachment of the retina [5,7,6,9] and possible increases in IOP in a percentage of cases varying from 20% to 80% [7,6,34,35]. Finally, the intravitreal administration of corticosteroids is only effective for a few months [36], which means that it is necessary to repeat the injections at three-monthly intervals to maintain stability of the retinal macula.

The subtenon TA administration has already been used in the treatment of cystoid macular edema and intermediate uveitis [10,11]. This administration route is not considered ideal to obtain a therapeutic dose of cortisone at the level of the retina [37] although this opinion can be contested on the basis of the clinical results and the ultrasound investigations which demonstrate how a correct administration of the injection makes it possible to deposit the drug in the macular area [38-40].

The subtenon approach is clearly less invasive than the intravitreal one [39] although, here again, this commonly used method is not free of potential complications such as the accidental injection directly into the choroidal or retinal circulation, perforation of the ocular bulb, occlusion of the central retinal artery and cataract [39]. Other complications described are blepharoptosis, orbital fat atrophy, strabismus and conjunctival necrosis [39,41]. IOP is not increased by the use of this approach with the exception of steroid responder patients [39,41].

This study has attempted to simplify the subtenon injection technique even further by using a 27 gauge needle, generally used for parabulbar anesthetics in cataract surgery. This approach made it possible to administer the injection without having to create a surgical opening in the conjunctiva to access the subtenon space, thus improving patient compliance with this therapy. When we use the subtenon approach for TA injection it is very important to make a careful echographic examination to determine the correct location near the macula of the drug. Without echography we cannot determine whether an unsatisfactory therapeutic response is secondary to the disease process or to misdisplacement of the TA. We think that our good results with the SBT approach in this study is related to the correct placement of TA near the macular area displayed with the echographic images.

## Conclusion

This study, although involving a limited number of selected patients, indicates how the eyes given a subtenon injection benefited from a more prolonged therapeutic efficacy of triamcinolone.

The subtenon approach, when the triamcinolone acetonide is correctly placed in the subtenon spaces, can be considered an easy, safe and valid alternative to the intravitreal injection.

## Competing interests

The author(s) declare that they have no competing interests.

## Authors' contributions

MC recruited the patient from the Retina Disease Service of the S. Orsola-Malpighi Hospital, he drafted the manuscript and performed the subtenon TA injection. AP performed the intravitreal TA injection. EZ reviewed the literature, PL examined the patient in the time and ECC review the manuscript. All authors read and approved the final manuscript.

## Pre-publication history

The pre-publication history for this paper can be accessed here:


